# *Aronia melanocarpa* (Black Chokeberry) Reduces Ethanol-Induced Gastric Damage via Regulation of HSP-70, NF-κB, and MCP-1 Signaling

**DOI:** 10.3390/ijms18061195

**Published:** 2017-06-05

**Authors:** Antonisamy Paulrayer, Aravinthan Adithan, Jeong Ho Lee, Kwang Hyun Moon, Dae Geun Kim, So Yeon Im, Chang-Won Kang, Nam Soo Kim, Jong-Hoon Kim

**Affiliations:** 1 College of Veterinary Medicine, BK21PLUS project, Chonbuk National University, 79 Gobong-ro, Iksan-city, Jeollabuk-do 54596, Korea; evolutionantony@gmail.com (A.P.); aravinthchandra@gmail.com (A.A.); cwkang@chonbuk.ac.kr (C.-W.K.); namsoo@jbnu.ac.kr (N.S.K.); 2 Sunchang Reserch Institute of Health and Longevity, Ingye-myeon Indeok-ro, Sunchang-gun, Jeollabuk-do 56015, Korea; wooju0717@hanmail.net (J.H.L.); khmoon@korea.kr (K.H.M.); roofdg@hanmail.net (D.G.K.); soyoun5005@hanmail.net (S.Y.I.)

**Keywords:** gastroprotective activity, ethanol-induced gastric ulcer, *Aronia melanocarpa*, ulcer index

## Abstract

*Aronia melanocarpa* (Michx.) Ell. belongs to the Rosaceae family. The purpose of this study is to explore the gastroprotective effect of the *Aronia melanocarpa* hydro-alcoholic extract (AMHAE) against ethanol-induced gastric ulcer in a rat model. Different concentrations (50, 100, and 200 mg/kg) of AMHAE, or 30 mg/kg of omeprazole, significantly inhibited the gastric injury formation. The ethanol-induced ulcer group showed significant increases of malondialdehyde (MDA), myeloperoxidase (MPO), tumor necrosis factor (TNF)-α, nuclear factor-kappaB p65 (NF-κB p65), and monocyte chemoattractant protein (MCP)-1, and decreased activities of superoxide dismutase (SOD), catalase (CAT), glutathione peroxidase (GSH-px), and interleukin (IL)-4. However, AMHAE (200 mg/kg) pretreatment significantly reversed the altered pathophysiological levels of these biomolecules to near normal stages. The gastroprotective activity of AMHAE was abolished by pretreatment with l-NAME, naloxone, capsazepine, and indomethacin, demonstrating the participation of nitric oxide (NO), opioids, TRPV (vanilloid receptor-related transient receptor potential), and prostaglandins in AMHAE-assisted gastroprotection against ethanol-induced gastric injuries. This gastroprotective effect of AMHAE might be due to the downregulation of TNF-α-based NF-κB, MCP-1 signaling and strong antioxidant properties.

## 1. Introduction

Gastric ulcer is a familiar gastrointestinal illness experienced globally. An acute gastric ulcer is frequently initiated by extreme intake of alcohol or a heavy doses of nonsteroidal anti-inflammatory drugs (NSAIDs) [1,2]. There is proof that a higher concentration of pure ethanol can lead to human gastric ulcers within 30 min of intake [3]. Gastrointestinal mucosa absorbs ethanol without any trouble. Ethanol not only induces direct injury to gastric mucosa, but also sensitizes the mucosa to injury during its short contact period with the gastric mucosa [4]. Ethanol-induced gastric injuries can appear within 30 min, and reach a peak level after 1 h. Excess intake of some alcoholic drinks can also create acute gastric damage [5]. While various studies focused on alcohol prompted gastric mucosal damage, the essential machineries are quite unclear.

In recent times, it has been revealed that ethanol-induced lipid peroxidation and oxidative stress has a major role in the pathogenesis of acute gastric injuries [4,6]. The production of reactive oxidative species (ROS) in gastric mucosal tissue continues at a normal level, due to the equilibrium between pro-oxidant and antioxidant systems. Conversely, the equilibrium is altered in many circumstances, including drinking alcohol [4].

Historically, black chokeberry (*Aronia melanocarpa*) originates from the eastern part of North America. At present the plant is cultivated nearly all over the world, including Korea; however, it has increased to the highest levels of popularity in European countries [7]. Properties of black chokeberries are extensively documented, and growing numbers of preclinical and clinical experiments have been directed to support the positive activities of *A. melanocarpa. A. melanocarpa* extracts and juices show anticancer, antihypertensive, cardioprotective, anti-inflammatory, antidiabetic, and immunomodulatory activities in animal and cellular models [8,9,10,11,12].

Fruit juice of *A. melanocarpa* showed gastroprotective activity against an indomethacin-induced ulcer model in rats [13]. The similar anti-ulcer activity of anthocyanin fractions from *A. melanocarpa* was recognized in ethanol-induced ulcers in rats [14]. Previous reports also elucidated the gastroprotective activities of other common berries, such as strawberries and *Rubus* berries, with similar gastroprotective effects against different ulcer models in animals. Strawberry extracts display a significant gastroprotective activity against ethanol-induced gastric injury, possibly associated to their anthocyanin content [15]. *Rubus* berry-derived polyphenols and anthocyanins showed considerable gastroprotective activities against different ulcer models in animals [16,17].

Yet, the defensive effect of *A. melanocarpa* has not yet been studied in any ulcer model in detail. Hence, the objective of the current experiment was to examine the gastroprotective effect of hydro-alcoholic extract of *A. melanocarpa* fruits against ethanol-induced gastric injury in rats in order to lead a reasonably harmless alternate for the conventional anti-ulcer drugs.

## 2. Results

**Figure 1:**
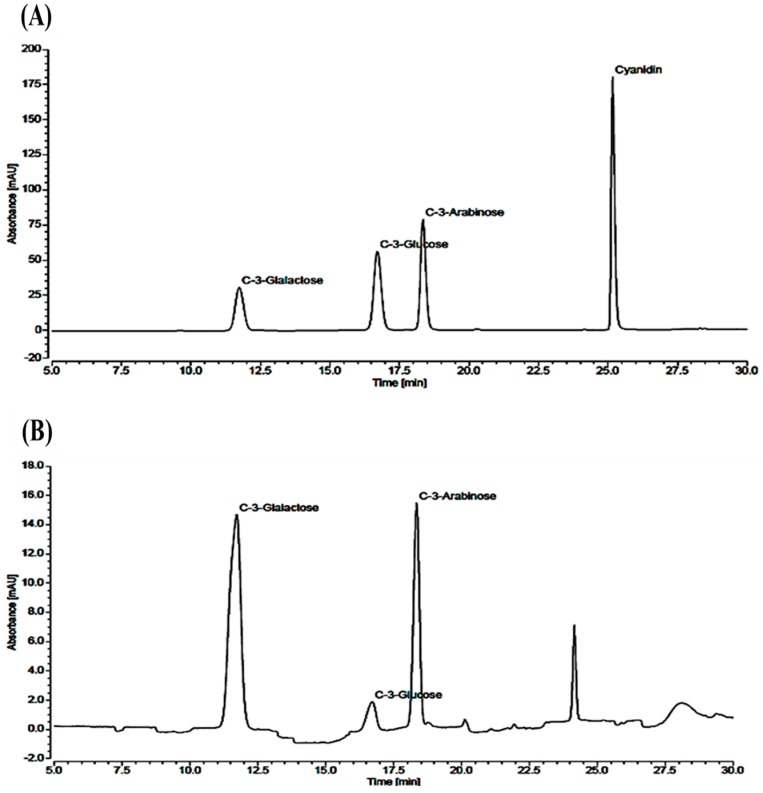
HPLC chromatograms of (**A**) standard and (**B**) sample. Peaks: cyanidin-3-galactose, cyanidin-3-glucose, and cyanidin-3-arabinose.

The total content of anthocyanin in the black chokeberry extract was 131.53 ± 0.51 mg/g (Table 1). Further, we examined the contents of cyanidin-3-galactose, cyanidin-3-glucose, and cyanidin-3-arabinose using the high-performance liquid chromatography (HPLC) method (Figure 1). The levels of cyanidin-3-galactose, cyanidin-3-glucose, and cyanidin-3-arabinose in black chokeberry extract were 79.31 ± 0.40, 10.16 ± 0.03, and 38.86 ± 0.66 mg/g, respectively (Table 2).

**Figure 2:**
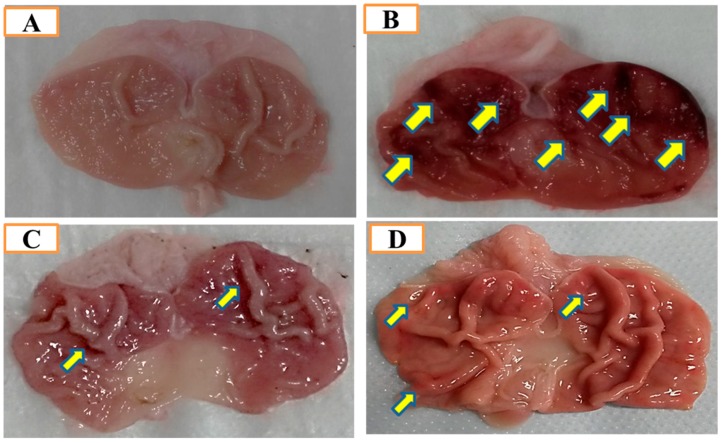
Gastroprotective activity of *Aronia melanocarpa* hydro-alcoholic extract (AMHAE) (200 mg/kg) on ethanol-induced gastric injury in rats. (**A**) Normal rats; (**B**) Ulcer control; (**C**) Omeprazole (30 mg/kg) pretreated rats; (**D**) AMHAE (200 mg/kg) pretreated rats. Ethanol-induced severe injuries to the gastric mucosa appear as elongated bands of hemorrhage (yellow arrows).

**Figure 3:**
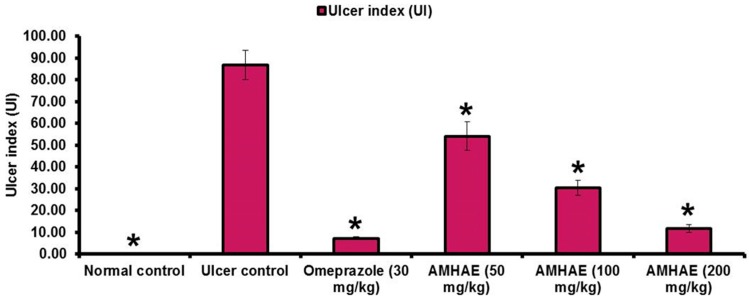
Effect of AMHAE (50, 100, and 200 mg/kg, orally) on the ethanol-induced ulcer index in rats. Values are mean ± S.D. (*n* = 6). * *p* < 0.05, comparing the ulcer control with all of the groups.

Macroscopic results displayed that the *Aronia melanocarpa* hydro-alcoholic extract (AMHAE) pretreated group (Figure 2D) or omeprazole group (Figure 2C) comprehensively reduced gastric wound compared to the ethanol-induced ulcer control group (Figure 2B). The normal control group displays an intact stomach devoid of any injuries (Figure 2A). AMHAE at the concentrations of 50, 100, and 200 mg/kg significantly inhibited the UI (ulcer index) by 37.59%, 65.07%, and 86.37%, respectively, as compared to the ethanol-induced ulcer control group. Similarly, omeprazole (30 mg/kg) also delivered significant gastroprotective effect by 91.65% (Figure 3). In assessment with the ulcer control group, the 200 mg/kg dose of AMHAE showed substantial ulcer protective activity compared with 50 and 100 mg/kg. Therefore, 200 mg/kg dose of AMHAE was chosen as the effective dose for further studies.

**Figure 4:**
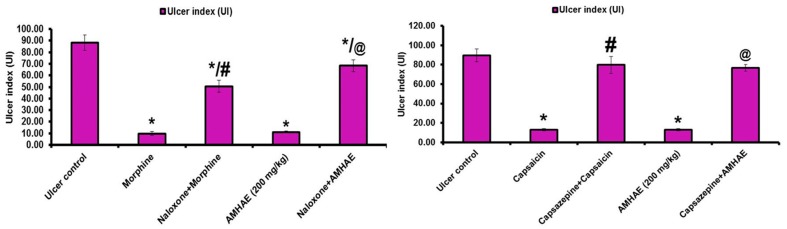

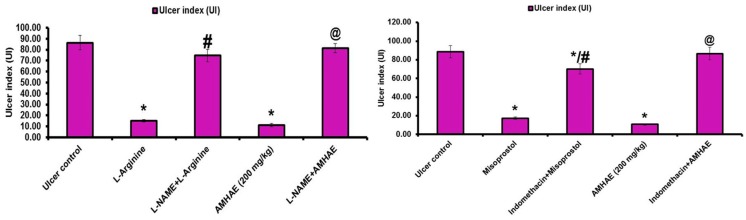
The effect of pretreatment with naloxone, capsazepine, *N*-G-nitro-l-arginine methyl ester (l-NAME), and indomethacin in gastroprotection provided by AMHAE. Values are mean ± S.D. (*n* = 6). * *p* < 0.05, comparing the ulcer control with all of the groups; # *p* < 0.05, comparing morphine with naloxone+morphine, capsaicin with capsazepine+capsaicin, l-arginine with l-NAME + l-arginine, or misoprostol with indomethacin+misoprostol; @ *p* < 0.05, comparing AMHAE with naloxone + AMHAE, capsaizepine + AMHAE, l-NAME + AMHAE, or indomethacin + AMHAE.

**Figure 5:**
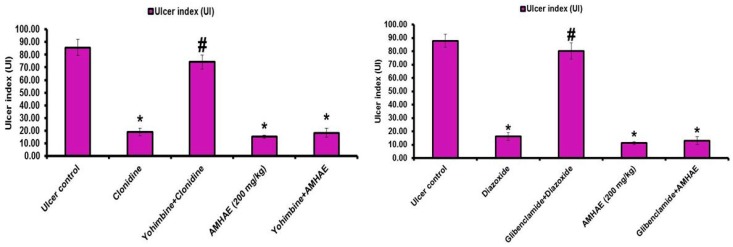
The effect of pretreatment with yohimbine and glibenclamide in gastroprotection provided by AMHAE. Values are mean ± S.D. (*n* = 6). * *p* < 0.05, comparing the ulcer control with all of the groups; # *p* < 0.05, comparing clonidine with yohimbine + clonidine or diazoxide with glibenclamide + diazoxide.

**Figure 6:**
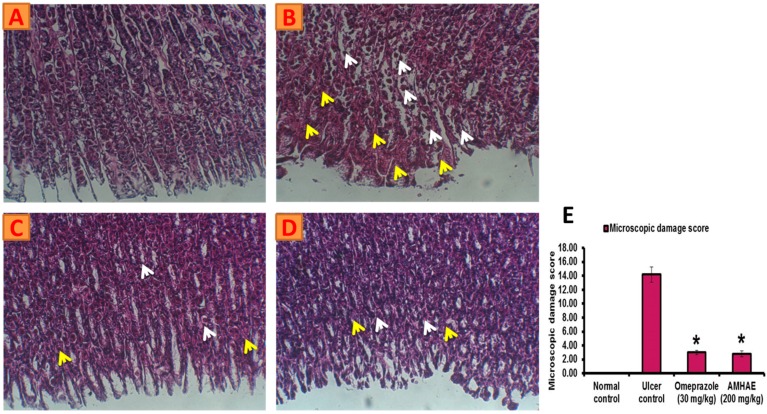
AMHAE (200 mg/kg) alleviates ethanol-induced gastric histological alterations in rats. (**A**) Normal; (**B**) ulcer control; (**C**) omeprazole (30 mg/kg); (**D**) AMHAE (200 mg/kg); and (**E**) microscopic damage score level of different groups. Original magnification 20×. * *p* < 0.05 compared ulcer control with all of the groups. Note: inflammatory cell invasion (white arrows) and Oedema formation (yellow arrows).

Treatment with naloxone, capsazepine, l-NAME, and indomethacin significantly (*p* < 0.05) inhibited the gastroprotective effect of AMHAE (200 mg/kg) by 65.05%, 71.20%, 81.26%, and 85.66%, respectively. However, treatment with yohimbine and glibenclamide did not alter AMHAE activity (Figure 4 and Figure 5). Gastric sections from the normal control group exhibited an undamaged architecture of the gastric tissue (Figure 6A). In contrast, the ethanol-induced ulcer control group showed serious gastric injury with a high level of microscopic impairment imitating hemorrhagic necrosis and interruption of the gastric mucosa with epithelial cell loss (Figure 6B). Pretreatment with AMHAE significantly reduced the pathologic scores that demonstrating the reduction of gastric injury and inflammatory cell infiltration with the protection of the stomach wall architecture (Figure 6D). These effects were equivalent to those given by the reference, omeprazole (Figure 6C). The microscopic damage score was significantly reduced by the treatment of AMHAE and omeprazole compared to ethanol-induced ulcer control group (Figure 6E).

**Figure 7:**
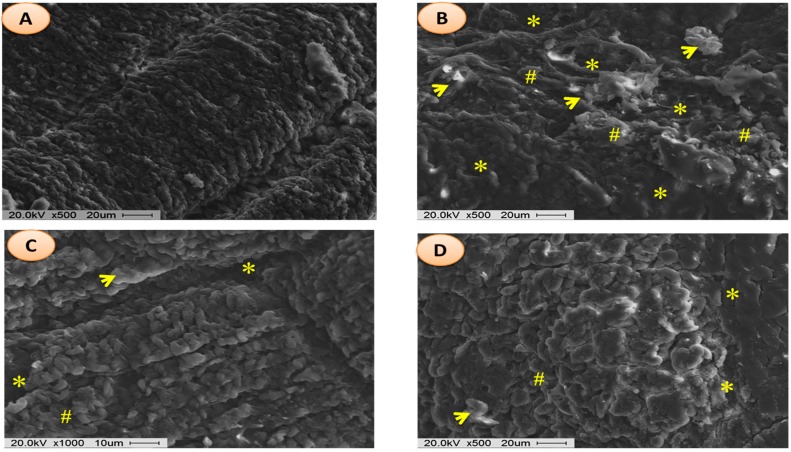
Scanning electron micrograph images of gastric injuries. Experimental groups: (**A**) normal; (**B**) ulcer control; (**C**) omeprazole (30 mg/kg); and (**D**) AMHAE (200 mg/kg). Note: epithelial desquamation (yellow asterisk); fibrin deposits and hemorrhage with erythrocytes (yellow arrows); and gastric epithelium with slightly widened gastric pits (pound signs).

**Figure 8:**
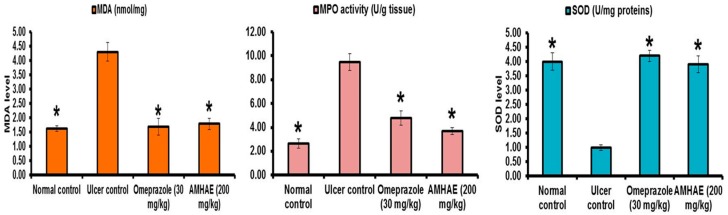

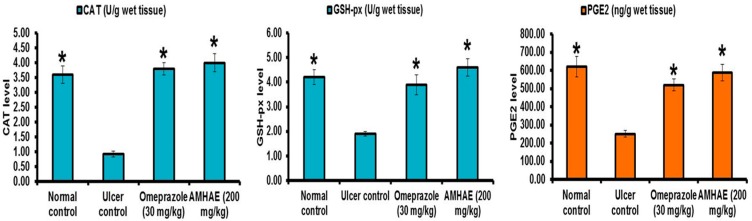
Effect of AMHAE (200 mg/kg, orally) on malondialdehyde (MDA), myeloperoxidase (MPO), superoxide dismutase (SOD), catalase (CAT), glutathione peroxidase (GSH-px), and prostaglandin E_2_ (PGE_2_) level. Values are mean ± S.D. (*n* = 6). * *p* < 0.05 compared ulcer control with all of the groups.

Scanning electron micrographs of rat fundic mucosa of various animal groups were depicted in Figure 7. Normal control group exhibited closely packed with gastric glands, the luminal surface of gastric epithelial cells and underlying muscularis mucosa (Figure 7A). Ethanol-induced ulcer control group showed the disturbed architecture of gastric mucosa, loss of surface epithelial cells with coverage of underlying lamina propria, and necrotic debris (Figure 7B). In the omeprazole and AMHAE pretreated group, scanning electron micrographs point out a nearly normal topography of gastric epithelium with marginally widened gastric pits and slight injury with small deposits of fibrin (Figure 7C,D). Malondialdehyde (MDA) and myeloperoxidase (MPO) levels were significantly (*p* < 0.05) elevated (2.63- and 3.59-fold, respectively) and the prostaglandin E_2_ (PGE_2_) level was decreased (2.47-fold) in the ethanol-induced ulcer control group. In opposition, treatment of AMHAE (200 mg/kg) showed a significant (*p* < 0.05) decline in MDA and MPO levels by 2.40- and 2.56-fold, respectively, and increased PGE_2_ level by 2.34-fold (Figure 8). Superoxide dismutase (SOD), catalase (CAT), and glutathione peroxidase (GSH-px) levels were significantly declined (4.0-, 3.90-, and 2.21-fold, respectively) in the ethanol-induced ulcer control group compared to the normal group. However, AMHAE (200 mg/kg) pretreated animals displayed a significant rise in SOD (3.90-fold), CAT (4.33-fold), and GSH-px (2.42-fold) levels compared to the ulcer control group (Figure 8).

**Figure 9:**
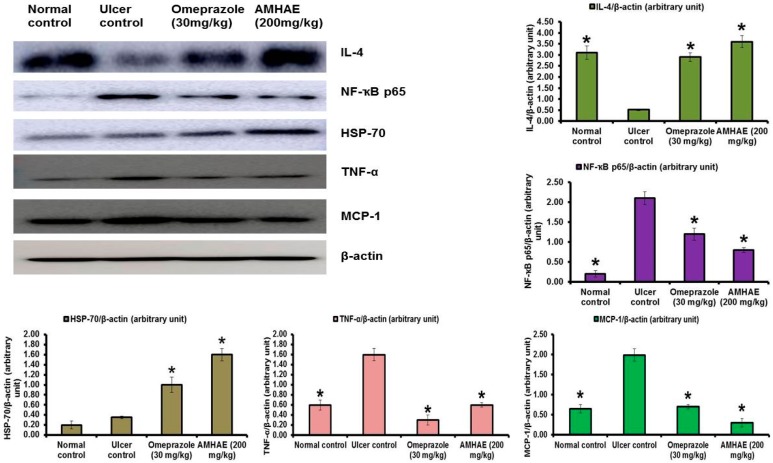
Effect of AMHAE (200 mg/kg) on the protein expression level of interleukin (IL)-4, nuclear factor-kappaB (NF-κB) p65, heat-shock protein-70 (HSP-70), tumor necrosis factor (TNF)-α, and monocyte chemoattractant protein (MCP)-1 in gastric mucosa. Levels of proteins of interest were normalized to the level of β-actin. Data were expressed as mean ± SD (* *p* < 0.05 when compared to the ethanol group).

Immunoblotting results confirmed that the ethanol-induced ulcer control group exhibited significant reductions in IL-4 (5.96-fold) level and increases of NF-κB p65 (10.50-fold), TNF-α (2.66-fold), and MCP-1 (3.06-fold) levels compared to the normal group. However, AMHAE pretreatment displayed significant increases of IL-4 (6.92-fold) and HSP-70 (4.57-fold) levels and a decline of NF-κB p65 (2.62-fold), TNF-α (2.66-fold), and MCP-1 (6.63-fold) levels compared to the ethanol-induced ulcer control group (Figure 9).

## 3. Discussion

Pre-synaptic α2-receptors facilitate various reactions in the digestive tract, and they participate in the regulation of acid secretion in the gastrointestinal tract [18]. Pretreatment with yohimbine (α2-receptors antagonist) did not block the gastroprotective effect of AMHAE against ethanol-induced gastric damage. In the same way, pretreatment of glibenclamide (inhibitor for ATP-sensitive potassium channel (K^+^ATP channel)) failed to block the protective effect of AMHAE against ethanol-induced gastric damage. Since yohimbine and glibenclamide were quiet in eliminating the AMHAE-delivered gastroprotection, we conclude that mechanisms excluding α2-receptors and the K^+^ATP channels contribute to its protective activity. l-NAME (non-selective inhibitor of nitric oxide synthase) efficiently blocked the gastroprotection caused by AMHAE, proposing NO contribution in its gastroprotection. It is well-identified that NO is a contributor in the inflection of gastric mucosal integrity, gastric mucosal blood flow, and mucus secretion [4,19]. In order to validate the starring role of prostaglandins in the gastroprotection provided by AMHAE, animals were pretreated with indomethacin (non-selective cyclooxygenase inhibitor). The outcomes exposed that the gastroprotection made by AMHAE against ethanol-induced gastric wounding was significantly alleviated by indomethacin, signifying a major part of endogenous prostaglandins in its gastroprotection. Treatment with capsazepine (vanilloid receptor antagonist) reduced the gastroprotective activity of AMHAE or capsaicin, indicating the possible contribution of primary afferent receptors sensitive to capsaicin. Vanilloids facilitate the production of NO through eNOS activation present in afferent neurons, contributing further to the gastroprotective effect [20]. Opioids and opiates considerably affect a range of digestive functions, including motility, secretion, and fluid transport by means of the stimulation of the opioid receptors [21]. The current experimental results displayed a reversal of the gastroprotective effect of AMHAE by the treatment of naloxone. This outcome highlights the association of opioid receptors in the protective mechanism of AMHAE [22].

Activation of nuclear factor-kappaB (NF-κB) is a vibrant pathophysiological process during ulcer formation, which stimulates pro-inflammatory cytokines, such as TNF-α and IL-1β [23,24]. In the present study, the increased level of nuclear NF-κB p65 was detected in the ethanol-induced ulcer control group, while the AMHAE-pretreated group showed a significant decline of NF-κB p65 level. It is possible that AMHAE was able to inhibit NF-κB stimulation via stabilization of IκBα and hindering of IKK activity. Heat-shock protein-70 (HSP-70) is a sort of protective protein with different roles in biological systems; it is commonly expressed due to exposure to different drugs, heat sensation, and oxidative stress [25]. In this experiment, when compared to the ethanol-induced ulcer control group, AMHAE pretreated group showed a significant increase in the HSP-70 production. Former studies specified that HSP-70 can stabilize IκBα via the hindrance of IKK activation [26]. It is promising that the reduction in NF-κB correlated to AMHAE pretreatment may be due to its upregulation capability on HSP-70 expression. MPO is a crucial indicator of neutrophil infiltration in ulcer-induced injuries [27]. This enzyme is abundantly expressed by the neutrophils via the oxidation process [28]. In the current study MPO activity was substantially augmented in the ethanol-induced ulcer control group, confirming neutrophil infiltration in gastric mucosa. AMHAE pretreatment significantly lowered MPO levels in ulcerated animals, proposing its capability to inhibit neutrophil infiltration in injured gastric tissue. These outcomes were in agreement with previous reports [4,19].

Lipid peroxidation is consequence of ROS reaction against cell membrane and produces significant levels of MDA, which leads to oxidative gastric damage [29]. In the present study, ethanol exposure causes significant rises of MDA level, and reductions in SOD, CAT, and GSH-px activities. Conversely, AMHAE treatment displays significant rises of SOD, CAT, and GSH-px, and a decrease of the MDA level, specifying its antioxidant potential. It is probable that direct antioxidant and free radical scavenging roles or increases of intracellular SOD, CAT, and GSH-px events in gastric tissues were responsible for AMHAE-assisted protective activity against ethanol-induced gastric ulcers, which is consistent with an earlier report that *Aronia melanocarpa* showed significant increases in free radical scavenging activity, antioxidant capability, and decreases of lipid peroxidation [30]. TNF-α showed multifaceted function during gastric ulcer formation and activated NF-κB, iNOS, and neutrophil infiltration. However, treatment of AMHAE exhibited a reduction of TNF-α and an increase of anti-inflammatory cytokine IL-4 revealed the anti-inflammatory nature of AMHAE.

Residential macrophages gathered in the interstitial space of the ulcer wound expressed higher quantities of MCP-1 and facilitated neutrophil and macrophage infiltration into the interstitial space [31]. In the present study AMHAE treatment significantly reduced the MCP-1 level and indicated the inhibition of neutrophil infiltration in the injured site. *Aronia melanocarpa* constitute important phytochemicals, such as polyphenols (including proanthocyanidins, anthocyanins, and hydroxycinnamic acids) and sugars [30]. Together with polyphenols, aronia berries also contain other bioactive components, including tannins, vitamins, bioelements, carotenoids, pectins, bioactive carbohydrates, organic acids, and proteins, but they are present in smaller amounts than polyphenolic constituents [30]. The plants with significant polyphenolic compounds possess considerable gastroprotective properties. These ingredients protect the gastrointestinal mucosa from injuries made by various necrotic agents [13].

It was proposed that 2 g/kg body weight of fruit extract of the black chokeberry and its red pigment fraction at 300 mg/kg showed nearly the same antiulcer activities and finally suggested that almost all of the antiulcer effects of the black chokeberry fruit extract could be obtained due to its red pigment fraction composed of cyanidin derivatives [14]. In agreement with this report, we proposed that gastroprotective activity of AMHAE was due to its major anthocyanin contents, such as cyanidin-3-galactose, cyanidin-3-glucose, and cyanidin-3-arabinose and its presence was verified via HPLC analysis. Previous reports also elucidated the protective activities of anthocyanins from strawberries and *Rubus* berries against different ulcer models in rodents [15,16,17]. Hence, it is possible that the gastroprotective activity of the AMHAE from *A. melanocarpa* is mainly due to its anthocyanin contents.

## 4. Materials and Methods

### 4.1. Animals

Male Sprague–Dawley (SD) rats (200–220 g) were used for this experimental study. Animals were kept at 23 ± 2 °C for 12 h light–dark cycles with 65–80% relative humidity and nourished with a regular pellet diet (Samyang, Daejeon, Korea) and water ad libitum. Rats were well-maintained in agreement with the guidelines distributed by the National Institute of Health for the Care and Use of Laboratory Animals (NIH Publication 80-23, revised in 1996). All of the animal experiments were conducted in accordance with the Ethics Committee norms (permit number CBNU-047, 2015) recognized by the Institutional Animal Care and Use Committee at Chonbuk National University (Jeonju, Korea).

### 4.2. Chemicals and Drugs

Omeprazole, glibenclamide (K^+^ATP channel inhibitor) *N*-G-nitro-l-arginine methyl ester (l-NAME; non-specific nitric oxide synthase inhibitor), carboxymethyl cellulose (CMC), l-arginine, clonidine, morphine, capsaicin, misoprostol, diazoxide, yohimbine, naloxone, capsazepine, indomethacin, cyanidin-3-galactose, cyanidin-3-glucose, and cyanidin-3-arabinose were obtained from Sigma-Aldrich (St. Louis, MO, USA). Ethanol was acquired from Merck (Darmstadt, Germany). Assay kits including SOD and CAT (DoGen, Seoul, Korea), MPO (Hycult Biotech, Uden, The Netherlands), MDA (BioVision, Milpitas, CA, USA), and PGE_2_ (R&D Systems, Minneapolis, MN, USA) were used for present examinations. 

### 4.3. Preparations of Hydro-Alcoholic Extract of Aronia melanocarpa

Preparations of *Aronia melanocarpa* hydro-alcoholic extract (AMHAE) are as follows: The black chokeberry fruits were collected from an authorized person in Cheongjeong-ro, Ssangchi-myeon, Sunchang-gun, Jeollabuk-do, Korea. A voucher specimen has been placed at the Chonbuk National University herbarium. The black chokeberry fruits were dried at room temperature and milled with an electric blender. The dried fruit powder (600 g) was soaked in 70% ethanol for 24 h at room temperature with concomitant shaking. Then, the extracts were filtered through Whatman filter paper (No. 2) and the filtrate was evaporated to remove the organic solvent under reduced pressure at a temperature less than 40 °C with a rotary evaporator (RE 200; Yamato Co., Tokyo, Japan). The crude extract was dried at 37 °C to complete the removal of the organic solvent. The final crude extraction yield was 36.66%.

### 4.4. Ethanol-Induced Gastric Injury

Test animals were fasted for 24 h and allocated into six groups (*n* = 6, respectively) as follows: normal and ulcer control groups received the vehicle (0.5% CMC), while the remaining groups received AMHAE (50, 100, and 200 mg/kg p.o.) and omeprazole (30 mg/kg p.o.). All of the above-stated drugs were given with 0.5% CMC as a vehicle. After 30 min, each animal group orally received 96% ethanol (5 mL/kg), excluding the normal group [19]. After 1 h, all rats were sacrificed under ether anesthesia condition and stomach samples were opened through greater curvature to observe the injury level macroscopically [32]. The percentage of ulcer index (UI) inhibition was calculated as follows:

[(UI_nontreated_ − UI_treated_)/UI_nontreated_] × 100

### 4.5. Preparation of Samples for Biochemical Analysis

Tissue samples were washed by using ice-cold saline. A tissue homogenate (10%) was prepared on ice with PBS (phosphate-buffered saline) buffer (50 mM phosphate buffer, pH 7.4) having a mammalian protease inhibitor cocktail. The homogenate was centrifuged for 10 min time duration at 4000× *g* at 4 °C. The resulting supernatant was used to quantify the various biochemical markers.

### 4.6. Biochemical Assays

SOD, CAT, GSH-px, MPO, MDA, and PGE_2_ levels were examined with relevant assay kits based on the manufacturer’s instructions. The activities are expressed as U/mg proteins (for SOD), U/g wet tissue (for CAT), U/g wet tissue (for GSH-px), U/g tissue (for MPO), and nmol/mg (for MDA).

### 4.7. Effects of l-NAME, Yohimbine, Naloxone, Capsazepine, Indomethacin, and Glibenclamide Pretreatments on the Gastroprotective Effect of AMHAE

The gastroprotective activity of AMHAE against ethanol-induced gastric injury was analysed through separate experiments in order to inspect the role of nitric oxide (NO), α2-receptors, opioid receptor, capsaicin-sensitive afferents, prostaglandins, and K^+^ATP channel activation. AMHAE (200 mg/kg) was administered along with the suitable agonists, l-arginine (600 mg/kg, i.p.), clonidine (0.05 mg/kg), morphine (5 mg/kg s.c.), capsaicin (5 mg/kg i.p.), misoprostol (0.16 mg/kg, p.o.), and diazoxide (10 mg/kg, p.o.), and the corresponding antagonists, l-NAME (20 mg/kg, i.p.), yohimbine (2 mg/kg, i.p.), naloxone (2 mg/kg i.p.), capsazepine (5 mg/kg i.p.), indomethacin (10 mg/kg, p.o.), and glibenclamide (5 mg/kg, p.o.), prior to the oral administration of 96% ethanol (5 mL/kg). In each circumstance, rats were pretreated with the exact antagonist 30 min before the application of AMHAE. After 1 h, the rats were sacrificed, the stomachs removed surgically, and opened along the greater curvature in order to observe the injury level macroscopically, as mentioned above.

### 4.8. Histopathologic Analysis and Microscopic Scoring of Gastric Injury

Gastric tissue was fixed in buffered 10% formalin for 24 h. Different tissue samples were washed and dehydrated via alcohol treatment and sectioned into small pieces and embedded by paraffin. Each paraffin block was sliced into 5 μm thicknesses and allowed to deparaffinize, followed by hematoxylin–eosin (H&E) staining, and inspected under a light microscope. A capable viewer unknowing about the specimens made all of the histopathologic scores in order to eliminate any bias. Gastric microscopic injury was scored (0–14 scale) based on a previous method [33]. A segment (1 cm long) of respective histological section was inspected for epithelial cell loss (score: 0–3), upper mucosa with edema (score: 0–4), hemorrhage (score: 0–4), and availability of inflammatory cells (score: 0–3).

### 4.9. Scanning Electron Microscopy Analysis of Gastric Injury

The stomach tissue was cut into small pieces and fixed in glutaraldehyde (2.5% in 0.1 M phosphate buffer) for 24 h. Each tissue sample was rinsed twice in phosphate buffer for 15 min followed by the post-fixative (1% OSO_4_ (osmium tetroxide) in 0.1 M phosphate buffer (pH 7.2)) for 2 h at room temperature. After post-fixation, tissue samples were rinsed with buffer for 15 min in order to eliminate unbound OSO_4_ out of the tissue. A dehydration process was then carried out in graded ethanol series for 15 min each. Tissue samples were then dried using a Baltec 030 critical point dryer (Natick, MA, USA) and coated with gold using a Baltec 030 sputter coater [34]. Investigation was carried out using a JEOL-JSM-6400 scanning electron microscope (Jeol Inc., Pleasanton, CA, USA). SEM handling and investigations were carried out at the electron microscopy unit, Faculty of Medicine Chonbuk National University (Jeonju, Korea).

### 4.10. Western Blot Analysis

Segments of gastric tissue were homogenized with radio immunoprecipitation assay (RIPA) buffer containing 1% Triton X-100, 1 mM EDTA, 10 mM Tris (pH 7.4), 1 mM EGTA, 150 mM NaCl, and protein inhibitor cocktail (1:10) (PhosSTOP ESAYpack, Roche, Basel, Switzerland). The total protein concentration was identified by using the Pierce™ BCA Protein Assay Kit (Pierce, Waltham, MA, USA) and 20 μg of proteins from different samples were electro-blotted onto a PVDF membrane followed by separation with 10% SDS-polyacrylamide gel electrophoresis. The immunoblot was then incubated with primary antibodies against IL-4, NF-κB p65, HSP-70, TNF-α, MCP-1, and β-actin (Abcam, Cambridge, UK). The obtained chemiluminescence signals were investigated with Adobe^®^ Photoshop CS3 and ImageJ software (http://rsb.info.nih.gov/ij/download.html).

### 4.11. Analysis of Major Anthocyanins by the HPLC Method

HPLC examination of black chokeberry extract was carried out based on a previous report [35]. Ten milligrams of crude extract were dissolved in 10 mL of water/formic acid (90/10, *v*/*v*, %) and filtered through a Millipore membrane filter (0.45 µm) before HPLC investigation. The HPLC apparatus was an Agilent 1260 with a photodiode array detector at 520 nm. The HPLC column was an Acclaim RSLC 120 (C18, 2 μm, 3.0 id × 100 mm, Agilent, Santa Clara, CA, USA) maintained at 35 °C. The mobile phase consisted of formic acid (10%) (A), and formic acid/methanol/acetonitrile (10/22.5/22.5, *v*/*v*/*v*, %) (B). The flow rate was 0.475 mL/min with the following gradient sequence: 0 min: 91% A + 09% B, 0.475 mL/min, 12 min: 91% A + 09% B, 0.475 mL/min, 25 min: 65% A + 35% B, 0.475 mL/min, 25 min: 50% A + 50% B, 0.475 mL/min, 30 min: 50% A + 50% B, 0.475 mL/min, 30 min: 91% A + 09% B, 0.475 mL/min, 35 min: 91% A + 09% B, 0.475 mL/min. The standards were cyanidin-3-galactose, cyanidin-3-glucose, and cyanidin-3-arabinose.

### 4.12. Determination of Total Anthocyanin Content

The amount of total anthocyanin content (TAC) was determined by the pH differential method based on AOAC, as mentioned by an earlier report [36]. The absorbance was detected at 510 and 700 nm in buffers with pH 1.0 and 4.5. The concentration of pigment was stated as mg cyanidin 3-glucoside equivalents/g dry mass and calculated with the following formula:

TA (mg/g) = *A* × 449.2 × DF × 103/26,900 × 1

where *A* = (*A*_520 nm_ − *A*_700 nm_)_pH 1.0_ − (*A*_520 nm_ − *A*_700 nm_)_pH 4.5_; MW (molecular weight) = 449.2 g/mol; DF = dilution factor; 103: factor to convert g to mg; 26,900: molar absorptivity of cyanidin-3-glucoside; 1: path length in cm.

### 4.13. Statistical Analysis

All experimental data were expressed as the mean ± standard deviation (SD) and analysed statistically via analysis of variance (ANOVA) and Tukey’s post hoc test. The probability value with *p* < 0.05 was considered significant.

## 5. Conclusions

In summary, the present study delivers considerable proof that the *Aronia melanocarpa* hydro-alcoholic extract (AMHAE) exhibited a significant gastroprotective role against ethanol-induced gastric injury in rats. The molecular mechanisms behind the gastroprotective effect of the AMHAE on ethanol-induced gastric ulcers in a rat model included a decline of inflammatory process (infiltration of inflammatory cells and oedema formation), reduction of MCP-1, MDA, NF-κB, and TNF-α levels, increases of antioxidants (SOD, CAT, GSH-px), and upregulation of IL-4, HSP-70, NO, and PGE_2_ expressions. Overall results shed new light on the effectiveness of aronia berries, which could appear to be worthy candidates for additional exploration of their prophylactic uses under ethanol-induced gastric ulcer conditions. However, additional research is necessary to elucidate the detailed mechanisms delivered by the AMHAE against ethanol-induced gastric ulcers.

## Figures and Tables

**Table 1:** Quantification of polyphenolic compounds in the black chokeberry extract.

Serial Number	Name of the Compound	Concentration (mg/g)
1	Total anthocyanins	131.53 ± 0.51
2	Cyanidin-3-galactose	79.31 ± 0.40
3	Cyanidin-3-glucose	10.16 ± 0.03
4	Cyanidin-3-arabinose	38.86 ± 0.66

Data is mean ± SD (*n* = 3).

**Table 2:** Analysis of anthocyanins by the high-performance liquid chromatography (HPLC) method.

Serial No.	Peak Name	Retention Time (min)	Area (mAU × min)	Height (mAU)	Relative Area (%)	Relative Height (%)
1	Cyanidin-3-galactose	11.720	6.986	14.634	60.53	45.94
2	Cyanidin-3-glucose	16.720	0.789	2.002	6.84	6.28
3	Cyanidin-3-arabinose	18.347	3.766	15.219	32.63	47.78
